# The application of clinical variable-based nomogram in predicting overall survival in malignant phyllodes tumors of the breast

**DOI:** 10.3389/fgene.2023.1133495

**Published:** 2023-05-30

**Authors:** Wei Li, Kun Fang, Jiaren Chen, Jian Deng, Dan Li, Hong Cao

**Affiliations:** ^1^ Department of Breast and Thyroid Surgery, The Second Affiliated Hospital, Hengyang Medical School, University of South China, Hengyang, China; ^2^ Department of Surgery, Yinchuan Maternal and Child Health Hospital, Yinchuan, China

**Keywords:** nomogram, SEER, prognosis, malignant phyllodes tumors, breast cancer

## Abstract

**Background:** We aimed to explore prognostic risk factors in patients with malignant phyllodes tumors (PTs) of the breast and construct a survival prediction model.

**Methods:** The Surveillance, Epidemiology, and End Results database was used to collect information on patients with malignant breast PTs from 2004 to 2015. The patients were randomly divided into training and validation groups using R software. Univariate and multivariate Cox regression analyses were used to screen out independent risk factors. Then, a nomogram model was developed in the training group and validated in the validation group, and the prediction performance and concordance were evaluated.

**Results:** The study included 508 patients with malignant PTs of the breast, including 356 in the training group and 152 in the validation group. Univariate and multivariate Cox proportional hazard regression analyses showed that age, tumor size, tumor stage, regional lymph node metastasis (N), distant metastasis (M) and tumor grade were independent risk factors for the 5-year survival rate of patients with breast PTs in the training group (*p* < 0.05). These factors were used to construct the nomogram prediction model. The results showed that the C-indices of the training and validation groups were 0.845 (95% confidence interval [CI] 0.802–0.888) and 0.784 (95% CI 0.688–0.880), respectively. The calibration curves of the two groups were close to the ideal 45° reference line and showed good performance and concordance. Receiver operating characteristic and decision curve analysis curves showed that the nomogram has better predictive accuracy than other clinical factors.

**Conclusion:** The nomogram prediction model constructed in this study has good predictive value. It can effectively assess the survival rates of patients with malignant breast PTs, which will aid in the personalized management and treatment of clinical patients.

## 1 Introduction

Breast phyllodes tumors (PTs) are rare fibroepithelial neoplasms that account for less than 1% of all breast tumors (Siegel et al., 2019). The World Health Organization classifies breast PTs as benign, marginal, or malignant based on their performance, prognosis, and treatment approaches. Malignant PTs are diagnosed when marked stromal hypercellularity, atypia, increased mitoses of ≥10/10 high-power fields (HPFs), permeative tumor borders, and stromal overgrowth are observed (Tan et al., 2016). The development of malignant PTs may be related to the loss of stromal dependency on the epithelium (Sawhney et al., 1992). Previous studies report that approximately 10%–15% of PTs are malignant, with poor prognostication and high recurrence and metastasis rates (Cimino-Mathews et al., 2013). For instance, about 15%–40% of malignant PTs recur locally, whereas 9%–27% metastasize to distant organs (Lissidini et al., 2022). Surgery is the primary treatment for PTs, and chemoradiotherapy is recommended for patients with recurring or metastatic tumors. However, increasing evidence shows that chemoradiotherapy is ineffective in PT patients with metastases and that these patients usually die within 3 year of treatment (Macdonald et al., 2006). Therefore, there is an urgent need to distinguish patients prone to recurrence/metastasis and provide effective treatment for patients with malignant PTs.

Nomogram is a visual and individualized predictive tool that provides a simple graphical representation of statistical predictive models to quantify personalized risk for a clinical event by incorporating a variety of risk factors (Balachandran et al., 2015). Nomograms are usually constructed for prognostic prediction and are widely used in many types of cancer because they can accurately predict patient survival time (Iasonos et al., 2008; Wang et al., 2013; Liang et al., 2015; Tan et al., 2018; Wu et al., 2018; Zhang et al., 2019). If the survival rate and severity of patients with malignant PTs can be accurately assessed by conventional clinical pathological characteristics, it can provide a high reference value for patients’ families and clinicians (Kim and Kim, 2017). Histological classification and tumor size are common risk factors for malignant PTs (Neron et al., 2020; Li et al., 2021; Choi et al., 2022). In addition, a study of 605 PT cases developed a nomogram based on histological criteria and surgical margins, in which stromal atypia, mitoses, overgrowth, and surgical margins (the AMOS criteria) were of independent significance in predicting the malignant behavior of PTs and the surgical margin status was considered the most important (Tan et al., 2012). However, determining which malignant PTs will undergo distant metastases remains difficult in clinical practice, and there are only a few studies on the prognostic prediction of malignant PTs.

In this study, based on the Surveillance, Epidemiology, and End Results (SEER) database, we developed a nomogram model to predict the overall survival of patients with malignant breast PTs, providing a basis for individualized diagnosis and treatment.

## 2 Methods

### 2.1 The source of the data

The data were published in the publicly available SEER database; therefore, all patient-identifying information was removed, and there was no need for approval from the Ethics Committee or informed consent from the patients. All data were approved by the National Institutes of Health before being obtained (approval number: 10934-Nov 2017). In this study, postoperative patient data on malignant PT-like breast tumors from 2004 to 2015 were obtained from the SEER database using the SEER-Stat 8.3.6 software.

### 2.2 Patient inclusion and information extraction

The study included the patients according to the following inclusion and exclusion criteria. The inclusion criteria were 1) malignant breast PTs; 2) breast cancer as the first primary malignant tumor; and 3) a follow-up duration of >1 month. The exclusion criteria were 1) unknown tumor grade; 2) lack of follow-up data; 3) unknown information about tumor-node-metastasis (TNM) stage; 4) unknown tumor size; 5) unknown race; and 6) presence of multiple primary tumors. The following information was extracted from 508 eligible patients: pathologically proven mammary phyllodes malignancies (ICD-O-3 9020/3), single-sided breast cancer, American Joint Committee on Cancer (AJCC) stage, patients diagnosed from 2004 to 2015, size of primary tumors (T), tumor grade, race, age, primary lesions, regional lymph node metastasis (N), distant metastasis (M), radiation therapy, and marital status. Although all 508 patients who met the group criteria underwent surgery, the specific details of the interventions were not provided in the SEER database; therefore, surgery could not be included as an independent prognostic variable in the model study.

**FIGURE 1:**
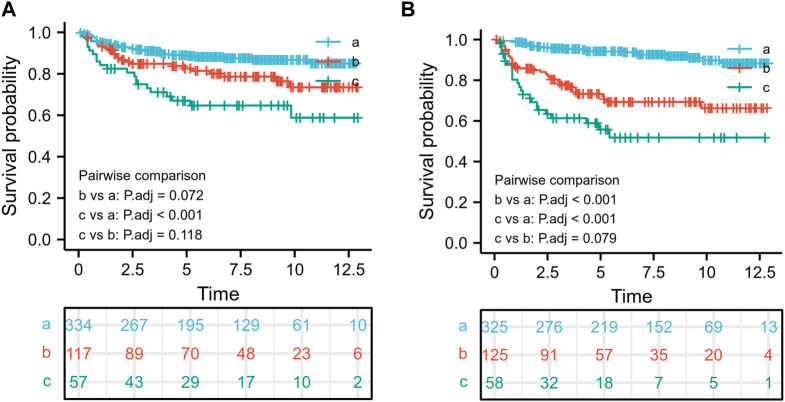
The optimal threshold for age and tumor size and the associated survival rate. The optimal threshold for age using the survival rate **(A)**,18–57 years old **(a)**, 57–67 years old **(b)**, and >67 years old **(c)**; The optimal threshold for tumor size using the survival rate **(B)**,<70 mm **(a)**, 70–160 mm **(b)**, and >160 mm **(c)**.

In the retrieved information, low-level tumors had high and moderate differentiation grades (ICD-O-3 1 and 2 levels), whereas high-level tumors had low or undifferentiated grades (ICD-O-3 3 and 4 levels). The critical values for diagnostic age and tumor size were determined using the X-tile software (Yale University, New Haven, CT, United States) (Camp et al., 2004), which has previously been shown to determine the critical point of the optimal tumor variable. The X-tile software was initially developed to determine the optimal threshold for breast cancer variables. The optimal threshold values for tumors were small (<70 mm), medium (70–160 mm), and large (>160 mm) (Figure 1). Because the optimal age limit was between 57 and 67 years (Figure 1), the patients were divided into three age groups (18–57, 57–67, or >67 years), as well as white, black, or other ethnic groups depending on race. Radiotherapy was divided into radiotherapy and non-radiotherapy. However, details such as radiotherapy type, site, and strength could not be obtained from the SEER database. Chemotherapy was divided into recipients and non-recipients.

### 2.3 Statistical analysis

Statistical analysis was conducted using the SPSS 25.0 software (IBM Corp., Armonk, NY, United States) and R language software (version 3.6.1, company, state, country). Patients were randomly divided into training (n = 356 cases) and validation (n = 152 cases) groups using R with a random sampling function. The univariate Cox regression analysis was performed to identify prognostic factors in the training group, and statistically significant variables (*p* < 0.05) were incorporated into a multivariate Cox risk regression model. Furthermore, the nomogram graph was constructed, with the C-index mapping the subject’s working receiver operating characteristic (ROC) curve and correction curve for decision curve analysis (DCA). In the correction curve, the closer the curve is to the ideal 45° guide, the closer the prediction is to the actual observation. The C-index and the area under the ROC curve (AUC) were used to evaluate the predicted value of the nomogram graph; the maximum C-index was 1 (representing 100% prediction accuracy), and the minimum value was 0.5 α. The closer the C-index was to 1, the better the accuracy of the prediction model. Using the DCA method, we could effectively determine the certainty and benefit of the prediction model. An α level of 0.05 was used.

## 3 Results

### 3.1 Patient baseline information

According to the inclusion and exclusion criteria, 508 patients with malignant breast PTs between 2004 and 2015 were screened out of the SEER database. Table 1 shows the key characteristics of patients with PTs. The patients were randomly divided into training (n = 356) and validation (n = 152) groups. The training group was used to construct the nomogram model, and the validation group was used to validate the model.

**TABLE 1:** Patients’ demographics and clinicopathological characteristics.

Variable	N	%
Entire	508	100
Age, y		
≤50	224	44.1
51–70	246	48.4
>70	38	7.5
Race		
White	369	78
Non-white	112	22
Laterality		
Left	254	50
Right	254	50
Grade		
Grade I	136	26.8
Grade II	117	23
Grade III	144	28.3
Grade IV	111	21.9
AJCC stage		
I-II	471	92.7
III-IV	37	7.3
T stage		
T1-T2	261	51.4
T3-T4	247	48.6
N stage		
N0	499	98.2
N1-N2	9	1.8
M stage		
M0	502	98.8
M1	6	1.2
Radiotherapy		
No	398	78.3
Yes	384	21.7
Chemotherapy		
No	488	96.1
Yes	20	3.9
Marital status		
Married	284	55.9
Unmarried	223	44.1

AJCC: American Joint Committee on Cancer; T: size of primary tumors; M: distant metastasis; N: regional lymph node metastasis.

### 3.2 Optimal tumor size and age truncation value

For further stratified analysis of age and tumor size, the X-tile graph determined the critical values of age to be 57 and 67 years, whereas the critical value of tumor size was determined to be 70 and 160 mm. To explore the effect of age-size thresholds on overall survival, we first divided the patients into three risk groups using 57 and 67 truncation values: 18–57 years old(a), 57–67 years old(b), and >67 years old(c). Furthermore, we found that age was an important prognostic factor in patients with malignant breast PTs; the worst prognostic factor for patients with malignant PTs was an age of >67 years, whereas patients aged <57 years had better overall survival rates than other groups (Figure 1A). Additionally, patients with a tumor size of >160 mm(c) had the worst prognosis, whereas those with a tumor size of <70 mm(a) had a better prognosis than the other groups (Figure 1B).

### 3.3 Prognostic factors for overall survival in patients with malignant PTs

To determine the independent prognostic factors for overall survival, we first performed univariate Cox regression analysis in the training group (n = 356). Factors such as older age, larger tumor size, race, tumor grade, chemotherapy, advanced AJCC stage, T stage, N stage, and distant metastasis (M) were significantly associated with poor overall survival (Table 2). These nine factors were further included and selected by the multivariate Cox proportional hazard regression analysis. These results showed that age at diagnosis, tumor grade, tumor size, N stage, and distant metastasis (M) were significant and independent prognostic factors for overall survival.

**TABLE 2:** Univariate and multivariate Cox regression analyses for overall survival in patients with malignant phyllode tumors of the breast.

	Univariate Cox				Multivariate Cox			
	HR	95%L	95%H	P	HR	95%L	95%H	P
Size								
<70 mm				0.000				0.000
70–160 mm	4.669	2.551	8.546	0.000	4.661	2.480	8.759	0.000
>160 mm	10.171	5.185	19.948	0.000	8.792	4.330	17.852	0.000
Age								
<58 y				0.000				0.000
58–67 y	1.504	0.800	2.827	0.205	1.426	0.745	2.728	0.284
>67 y	3.701	2.070	6.617	0.000	4.259	2.357	7.697	0.000
Race								
Black				0.067				
Other	0.516	0.204	1.308	0.163				
White	0.443	0.223	0.880	0.020				
Grade	3.555	1.987	6.360	0.000	2.309	1.268	4.205	0.006
Laterality	1.189	0.723	1.955	0.494				
Radiation	1.485	0.849	2.598	0.165				
Chemotherapy	2.999	1.291	6.966	0.011				
AJCC	6.465	3.582	11.669	0.000				
T	4.370	2.443	7.817	0.000				
N	9.608	3.802	24.285	0.000	4.478	1.538	13.034	0.006
M	49.191	16.285	148.587	0.000	30.673	8.421	111.729	0.000
Marital status	1.517	0.925	2.487	0.099				

AJCC: American Joint Committee on Cancer; T: size of primary tumors; M: distant metastasis; N: regional lymph node metastasis.

### 3.4 Construction and validation of the prognostic model for overall survival

**FIGURE 2:**
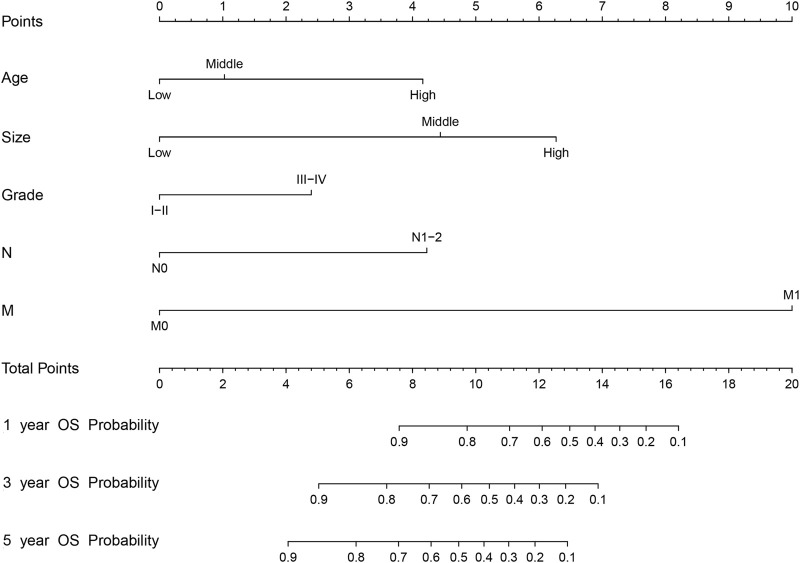
The nomogram model for predicting 1-, 3-, and 5-year OS (Overall Survival) rates of malignant breast PTs based on risk factors such as age, size, tumor grade, N stage, and distant metastasis (M). (Age-Age, Size-Size, and Grade-Level).

**FIGURE 3:**
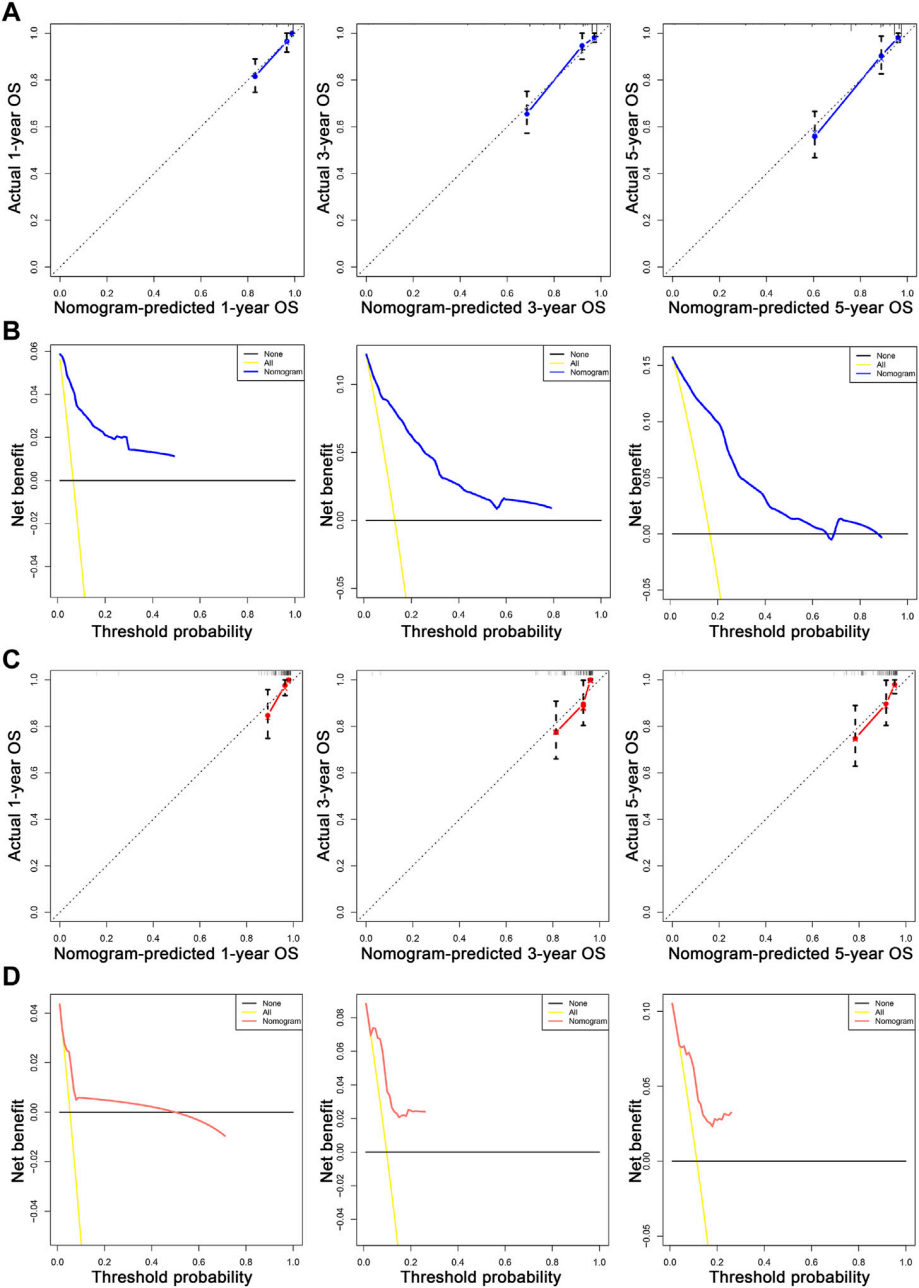
The calibration and DCA plots of the nomogram for predicting 1-, 3-, and 5-year OS. **(A, C)** The calibration plots for predicting 1-, 3-, and 5-year OS in the training and validation sets, respectively. **(B, D)** show the DCA curves evaluating the model’s clinical applicability in the training and validation sets, respectively. In the calibration curve, the X-axis is the OS predicted by the model. The actual OS is drawn on the Y-axis. The ideal calibration model is presented, in which the predicted probability corresponds to the actual survival outcome. In the decision curve, the X-axis represents a percentage of the threshold probability. In contrast, the Y-axis represents a net gain calculated by subtracting the true-positive number from the false-positive number.

We first combined age, tumor size, N stage, distant metastasis (M), and tumor grade to construct a prognostic nomogram model for predicting the 1-, 3-, and 5-year overall survival in patients with malignant breast PTs (Figure 2). The model can score each prognostication factor on the score scale. By combining these scores with the total score of the lowest score, the 1-, 3-, and 5-year overall survival predictions for patients with malignant PTs of the breast can be calculated. The predictive accuracy of the final prognostication model was evaluated by the C-index. The C-index was 0.845 (95% confidence interval [CI], 0.802–0.888) and 0.784 (95% CI, 0.688–0.880) in the training and validation groups, respectively. The calibration plots revealed perfect consistency between the model-predicted and actual survival values (Figures 3A, C). Surgeons can easily use these prognostic models to determine the prognosis of patients with malignant PTs: age, tumor size, N stage, distant metastasis (M), and tumor grade. 

**FIGURE 4:**
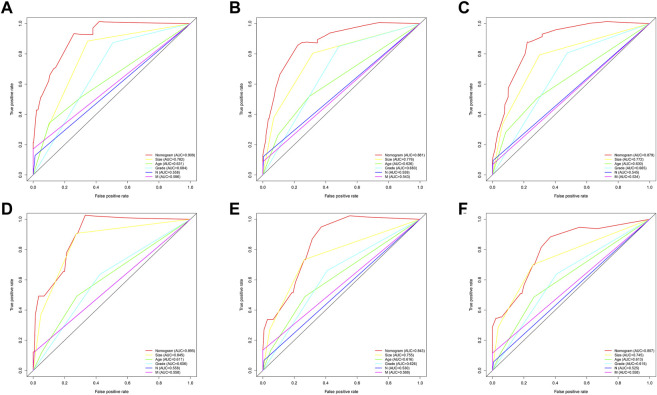
ROC curves for comparison of the sensitivity and specificity of the nomogram and clinical risk factors. The AUC comparison of the nomogram and clinical risk factors for predicting total annual survival rates at 1 **(A)**, 3 **(B)**, and 5 **(C)** years in the training group and **(D–F)** in the validation set, respectively.

Then, the predictive ability of the nomogram model was evaluated using the AUC (an AUC value of 0.5 indicates that the nomogram model has no predictive effect, and an AUC value of 1 indicates that the nomogram model has an excellent predictive effect). Patients with different survival rates were well distinguished (the higher the value between 0.5 and 1 is, the better the nomogram’s ability to distinguish), and other prognostic factors could be compared to further validate their superiority and determine which factor has the most superior predictive accuracy. In the training group, the advantages of the differentiation for overall survival shown in the nomogram were demonstrated compared to the other clinical information (1-year AUC: 0.908; 3-year AUC: 0.881; 5-year AUC: 0.879; Figures 4A–C). In addition, in the validation group, the AUC values for the 1-, 3-, and 5-year survival rates were 0.895, 0.843, and 0.807, respectively.

The nomogram offers a considerable positive net benefit from the risk of mortality in both the training and validation groups (Figures 3B, D). It can predict the 1-, 3-, and 5-year overall survival rates and has good value in clinical application. Using the nomogram model (Figure 2), we can predict a patient’s survival probability based on his/her personalized information. For example, in a middle-aged woman diagnosed with breast PTs with a medium-sized primary lesion and tumor grade III, the patient’s overall survival score was 10.5 according to the model. As a result, the patient’s 1-, 3-, and 5-year overall survival rates were estimated to be 90%, 82%, and 80%, respectively.

## 4 Discussion

Compared with previous studies, we developed a nomogram mainly for patients with malignant PTs in our study (Ma et al., 2021; Choi et al., 2022; Bedi et al., 2022; Zhou et al., 2018b). Our nomogram performed well in terms of discrimination and calibration, and surgeons can use it to evaluate prognosis using patient-specific clinical information.

A few clinical factors may affect the survival of patients with malignant breast PTs, but previous studies have not fully integrated clinical factors (Asoglu et al., 2004; Majeski and Stroud, 2012; Wang et al., 2015; Zhang and Kleer, 2016; Strode et al., 2017; Co et al., 2018). A prognostic nomogram model is a graphical representation of several variables that combines many risk factors to accurately assess survival probability at a given period (Jin et al., 2020; Wen-Tao et al., 2021). Prognostic assessment models have been established for certain cancers, and they have proven to be more accurate than traditional tools for prognostic prediction (Zhou et al., 2018a; Dong et al., 2018; Ye et al., 2018; Zheng et al., 2018). In recent years, few studies have focused on the prognostic prediction of malignant breast PTs. Previous studies have found that surgical methods and tumor boundaries are independent prognostic factors for recurrence in marginal and malignant PTs (Neron et al., 2020; Li et al., 2021; Choi et al., 2022).

Breast PTs are rare fibroepithelial neoplasms. A study demonstrated that metastasis was predicted in malignant PTs based on tumor size and the presence of malignant heterologous elements (Koh et al., 2018). A meta-analysis of 54 studies with 9234 cases suggested that mitoses, tumor boundary, stromal cellularity, stromal atypia, stromal overgrowth, tumor necrosis, surgery type, and surgical margin status are risk factors for local recurrence (LR) (Lu et al., 2019). However, given the low incidence of malignant breast PTs, no collaborative model for predicting prognosis was available. Singaporean scholars are also attempting to predict the clinical behavior of breast PTs. Line charts based on histological criteria and surgical cut edges are used to evaluate the prognostication of PTs (Asoglu et al., 2004; Majeski and Stroud, 2012; Zhang and Kleer, 2016; Strode et al., 2017).

This study used multivariate Cox proportional hazard regression analysis to determine the independent prognostic factors for OS, including patient age, tumor grade, N stage, presence of distant metastasis (M), and tumor size. Tumor grade, N stage, and distant metastasis (M) are widely accepted as significant risk factors for LR. Patients with LR and metastases have a worse prognosis. Among patients with malignant PTs, older patients had poor OS. Prior research on the effect of tumor size on prognosis yielded conflicting findings. Kapiris et al. (2001) reported that tumor size and excision are associated with LR of malignant breast PTs. Asoglu et al. (2004) demonstrated that excessive substrate growth, tumor size, and the excision cut edge are essential factors for LR. However, it is unclear whether tumor size is a predictor of LR, as some studies have found that tumor size is not associated with LR (Salvadori et al., 1989) and that LR does not affect survival, which are inconsistent with our findings. Our study found a significant correlation between larger tumor size and shorter OS in the multivariate analysis, possibly because a tumor’s biological characteristics are more aggressive and its state tends to be worse as it becomes larger. It has also been suggested that radiation therapy (RT) can improve patient survival by reducing the risk of LR. RT was excluded from our study because of limited data. Nevertheless, the validity of the study must be examined because one of the study events was disease-free survival rather than LR (Zeng et al., 2015).

This study has several limitations. Because the insignificance of Her-2 has only been documented in the SEER database since 2010, we avoided molecular subtype analysis of breast cancer and used AJCC stage and TNM stage analyses to reduce case loss. The findings will help with the external validation cohort in the future. Clinical data and prospective studies are required to validate the value of the nomogram.

## 5 Conclusion

Patient age, tumor grade, N stage, distant metastasis (M), and tumor size were found to be independent factors for overall survival in malignant PTs of the breast. Compared with prognostic factors alone, these integrated factors and nomograms, can be used as a valuable and convenient assessment tool to help surgeons personalize survival assessments and identify mortality risk in patients with malignant PTs of the breast.

## Data Availability

The original contributions presented in the study are included in the article/Supplementary Material, further inquiries can be directed to the corresponding authors.

## References

[B1] Asoglu O. Ugurlu M. M. Blanchard K. Grant C. S. Reynolds C. Cha S. S. (2004). Risk factors for recurrence and death after primary surgical treatment of malignant phyllodes tumors. Ann. Surg. Oncol. 11, 1011–1017. 10.1245/ASO.2004.02.001 15525831

[B2] Balachandran V. P. Gonen M. Smith J. J. DeMatteo R. P. (2015). Nomograms in oncology: More than meets the eye. Lancet Oncol. 16, e173–e180. 10.1016/S1470-2045(14)71116-7 25846097 PMC4465353

[B3] Camp R. L. Dolled-Filhart M. Rimm D. L. (2004). X-Tile: A new bio-informatics tool for biomarker assessment and outcome-based cut-point optimization. Clin. Cancer Res. 10, 7252–7259. 10.1158/1078-0432.CCR-04-0713 15534099

[B4] Choi J. E. Kang S. H. Tan P. H. Bae Y. K. (2022). Recurrence prediction for breast phyllodes tumours: Validation of the Singapore nomogram in Korean women. J. Clin. Pathol. 75, 159–163. 10.1136/jclinpath-2020-207093 33376198

[B5] Cimino-Mathews A. Hicks J. L. Sharma R. Vang R. Illei P. B. De Marzo A. (2013). A subset of malignant phyllodes tumors harbors alterations in the Rb/p16 pathway. Hum. Pathol. 44, 2494–2500. 10.1016/j.humpath.2013.06.009 23916291 PMC3998645

[B6] Co M. Chen C. Tsang J. Y. Tse G. Kwong A. (2018). Mammary phyllodes tumour: A 15-year multicentre clinical review. J. Clin. Pathol. 71, 493–497. 10.1136/jclinpath-2017-204827 29146885

[B7] Dong F. Shen Y. Gao F. Shi X. Xu T. Wang X. (2018). Nomograms to predict individual prognosis of patients with primary small cell carcinoma of the bladder. J. Cancer 9, 1152–1164. 10.7150/jca.23344 29675096 PMC5907663

[B8] Iasonos A. Schrag D. Raj G. V. Panageas K. S. (2008). How to build and interpret a nomogram for cancer prognosis. J. Clin. Oncol. 26, 1364–1370. 10.1200/JCO.2007.12.9791 18323559

[B9] Jin Y. Li Y. Liu Q. Li L. Feng A. Wang T. (2020). Brief introduction of medical database and data mining technology in big data era. J. Evid. Based Med. 13, 57–69. 10.1111/jebm.12373 32086994 PMC7065247

[B10] Kapiris I. Nasiri N. A'Hern R. Healy V. Gui G. P. (2001). Outcome and predictive factors of local recurrence and distant metastases following primary surgical treatment of high-grade malignant phyllodes tumours of the breast. Eur. J. Surg. Oncol. 27, 723–730. 10.1053/ejso.2001.1207 11735168

[B11] Kim Y. J. Kim K. (2017). Radiation therapy for malignant phyllodes tumor of the breast: An analysis of SEER data. Breast 32, 26–32. 10.1016/j.breast.2016.12.006 28013032

[B12] Koh V. C. Y. Thike A. A. Nasir N. D. M. Yip G. W. C. Bay B. H. Tan P. H. (2018). Size and heterologous elements predict metastases in malignant phyllodes tumours of the breast. J. .Virchows Arch. 472, 615–621. 10.1007/s00428-017-2257-1 29127495

[B13] Li Y. Song Y. Lang R. Shi L. Gao S. Liu H. (2021). Retrospective study of malignant phyllodes tumors of the breast: Younger age, prior fibroadenoma surgery, malignant heterologous elements and surgical margins may predict recurrence. Breast 57, 62–70. 10.1016/j.breast.2021.03.001 33774460 PMC8027899

[B14] Liang W. Zhang L. Jiang G. Wang Q. Liu L. Liu D. (2015). Development and validation of a nomogram for predicting survival in patients with resected non-small-cell lung cancer. J. Clin. Oncol. 33, 861–869. 10.1200/JCO.2014.56.6661 25624438

[B15] Lissidini G. Mule A. Santoro A. Papa G. Nicosia L. Cassano E. (2022). Malignant phyllodes tumor of the breast: A systematic review. Pathologica 114, 111–120. 10.32074/1591-951X-754 35414723 PMC9248247

[B16] Lu Y. Chen Y. Zhu L. Cartwright P. Song E. Jacobs L. (2019). Local recurrence of benign, borderline, and malignant phyllodes tumors of the breast: A systematic review and meta-analysis. Ann. Surg. Oncol. 26, 1263–1275. 10.1245/s10434-018-07134-5 30617873

[B17] Macdonald O. K. Lee C. M. Tward J. D. Chappel C. D. Gaffney D. K. (2006). Malignant phyllodes tumor of the female breast: Association of primary therapy with cause-specific survival from the surveillance, Epidemiology, and End results (SEER) program. Cancer 107, 2127–2133. 10.1002/cncr.22228 16998937

[B18] Majeski J. Stroud J. (2012). Malignant phyllodes tumors of the breast: A study in clinical practice. Int. Surg. 97, 95–98. 10.9738/CC79.1 23102073 PMC3723204

[B19] Neron M. Sajour C. Thezenas S. Piperno-Neumann S. Reyal F. Laé M. (2020). Surgical margins and adjuvant therapies in malignant phyllodes tumors of the breast: A multicenter retrospective study. Ann. Surg. Oncol. 27, 1818–1827. 10.1245/s10434-020-08217-y 31989361

[B20] Salvadori B. Cusumano F. Del Bo R. Delledonne V. Grassi M. Rovini D. (1989). Surgical treatment of phyllodes tumors of the breast. Cancer 63, 2532–2536. 10.1002/1097-0142(19890615)63:12<2532::aid-cncr2820631229>3.0.co;2-q 2541890

[B21] Sawhney N. Garrahan N. Douglas-Jones A. G. Williams E. D. (1992). Epithelial–stromal interactions in tumors. A morphologic study of fibroepithelial tumors of the breast. Cancer 70, 2115–2120. 10.1002/1097-0142(19921015)70:8<2115::aid-cncr2820700818>3.0.co;2-k 1327488

[B22] Siegel R. L. Miller K. D. Jemal A. (2019). Cancer statistics, 2019. CA Cancer J. Clin. 69, 7–34. 10.3322/caac.21551 30620402

[B23] Strode M. Khoury T. Mangieri C. Takabe K. (2017). Update on the diagnosis and management of malignant phyllodes tumors of the breast. Breast 33, 91–96. 10.1016/j.breast.2017.03.001 28327352

[B24] Tan B. Y. Acs G. Apple S. K. Badve S. Bleiweiss I. J. Brogi E. (2016). Phyllodes tumours of the breast: A consensus review. Histopathology 68, 5–21. 10.1111/his.12876 26768026 PMC5027876

[B25] Tan P. H. Thike A. A. Tan W. J. Thu M. M. M. Busmanis I. Li H. (2012). Predicting clinical behaviour of breast phyllodes tumours: A nomogram based on histological criteria and surgical margins. J. Clin. Pathol. 65, 69–76. 10.1136/jclinpath-2011-200368 22049216

[B26] Tan X. R. Li Y. Q. Liang S. B. Jiang W. Liu F. Ge W. X. (2018). Development and validation of a gene expression-based signature to predict distant metastasis in locoregionally advanced nasopharyngeal carcinoma: A retrospective, multicentre, cohort study. Lancet Oncol. 19, 382–393. 10.1016/S1470-2045(18)30080-9 29428165

[B27] Wang F. Jia Y. Tong Z. (2015). Comparison of the clinical and prognostic features of primary breast sarcomas and malignant phyllodes tumor. Jpn. J. Clin. Oncol. 45, 146–152. 10.1093/jjco/hyu177 25387733

[B28] Wang Y. Li J. Xia Y. Gong R. Wang K. Yan Z. (2013). Prognostic nomogram for intrahepatic cholangiocarcinoma after partial hepatectomy. J. Clin. Oncol. 31, 1188–1195. 10.1200/JCO.2012.41.5984 23358969

[B29] Wen-Tao W. Yuan-Jie L. Ao-Zi F. Li L. Huang T. Xu A. D. (2021). Data mining in clinical big data: The frequently used databases, steps, and methodological models. Mil. Med. Res. 8, 44. 10.1186/s40779-021-00338-z 34380547 PMC8356424

[B30] Wu S. Chen J. N. Zhang Q. W. Tang C. T. Zhang X. T. Tang M. Y. (2018). A new metastatic lymph node classification-based survival predicting model in patients with small bowel adenocarcinoma: A derivation and validation study. EBioMedicine 32, 134–141. 10.1016/j.ebiom.2018.05.022 29908920 PMC6021266

[B31] Ye F. G. Xia C. Ma D. Lin P. Y. Hu X. Shao Z. M. (2018). Nomogram for predicting preoperative lymph node involvement in patients with invasive micropapillary carcinoma of breast: A SEER population-based study. BMC Cancer 18, 1085. 10.1186/s12885-018-4982-5 30409127 PMC6225632

[B32] Zeng S. Zhang X. Yang D. Wang X. Ren G. (2015). Effects of adjuvant radiotherapy on borderline and malignant phyllodes tumors: A systematic review and meta-analysis. Mol. Clin. Oncol. 3, 663–671. 10.3892/mco.2015.503 26137284 PMC4471537

[B33] Zhang L. Dong D. Li H. Tian J. Ouyang F. Mo X. (2019). Development and validation of a magnetic resonance imaging-based model for the prediction of distant metastasis before initial treatment of nasopharyngeal carcinoma: A retrospective cohort study. EBioMedicine 40, 327–335. 10.1016/j.ebiom.2019.01.013 30642750 PMC6413336

[B34] Zhang Y. Kleer C. G. (2016). Phyllodes tumor of the breast: Histopathologic features, differential diagnosis, and molecular/genetic updates. Arch. Pathol. Lab. Med. 140, 665–671. 10.5858/arpa.2016-0042-RA 27362571

[B35] Zheng W. Huang Y. Chen H. Wang N. Xiao W. Liang Y. (2018). Nomogram application to predict overall and cancer-specific survival in osteosarcoma. Cancer Manag. Res. 10, 5439–5450. 10.2147/CMAR.S177945 30519092 PMC6235004

[B36] Zhou H. Zhang Y. Qiu Z. Chen G. Hong S. Chen X. (2018). Nomogram to predict cause-specific mortality in patients with surgically resected stage I non-small-cell lung cancer: A competing risk analysis. Clin. Lung Cancer 19, e195–e203. 10.1016/j.cllc.2017.10.016 29153966

[B37] Zhou Z. R. Wang C. C. Sun X. J. Yang Z. Z. Chen X. X. Shao Z. M. (2018). Prognostic factors in breast phyllodes tumors: A nomogram based on a retrospective cohort study of 404 patients. Cancer Med. 7, 1030–1042. 10.1002/cam4.1327 29479819 PMC5911599

